# Intra- and interobserver analysis in the morphological assessment of early stage embryos during an IVF procedure: a multicentre study

**DOI:** 10.1186/1477-7827-9-127

**Published:** 2011-09-15

**Authors:** Goedele Paternot, Alex M Wetsels, Fabienne Thonon, Anne Vansteenbrugge, Dorien Willemen, Johanna Devroe, Sophie Debrock, Thomas M D'Hooghe, Carl Spiessens

**Affiliations:** 1 Leuven University Fertility Centre, UZ Gasthuisberg, Leuven, Belgium; 2 Radboud University Nijmegen Medical Centre, Nijmegen, the Netherlands; 3 Centre de Procréation Médicalement Assistée de l'ULg, CHR de la Citadelle, Liège, Belgium; 4 Service PMA, Centre Hospitalier Régional de Namur, Namur, Belgium

## Abstract

**Background:**

Quality control programs are necessary to maintain good clinical practice. Embryo grading has been described as one of the external quality assurance schemes. Although the evaluation of embryos is based on the assessment of morphological characteristics, considerable intra- and inter-observer variability has been described. In this multicentre study, the variability in the embryo evaluation has been evaluated using morphological characteristics on day 1, day 2 and day 3 of embryo development.

**Methods:**

Five embryologists of four different IVF centers participated in this study. Multilevel images of embryos were presented on a website at different time points to evaluate intra-and inter-observer agreement in the assessment of embryo morphology. The embryos were evaluated on day 1, day 2 and day 3 of their development and each embryologist had to decide if the embryo had to be transferred, cryopreserved or discarded.

**Results:**

Both intra-observer agreement and inter-observer agreement were good to excellent for the position of the pronuclei on day 1, the number of blastomeres on day 2 and day 3 and the clinical decision (transfer, cryopreservation, discard). For all other characteristics (size of pronuclei, presence of cytoplasomic halo, degree of fragmentation and size of blastomeres) the intra- and inter-observer agreement was moderate to very poor.

**Conclusions:**

Mono- or multicentre quality control on embryo scoring by morphological assessment can easily be performed through the design of a simple website. In the future the website design can be adapted to generate statistical feedback upon scoring and can even include a training module.

## Background

The laboratory phase of in vitro fertilization (IVF) treatments consists of complex procedures, requiring high quality devices, equipment and personnel. The efficiency and effectiveness of these laboratories can be controlled by the application of strict norms (ISO 15189; 9001) in combination with clinical practice guidelines and internal and external quality control programs, as reported before [1].

Embryo grading has been proposed as one of the external quality assurance schemes [1] and is principally based on the assessment of morphological characteristics in a fast, easy and non-invasive way [2]. As stated by Racowsky et al., a grading system must be simple, containing characteristics with a proven predictive value and easily to adopt in different lab [3]. Therefore, the SART committee developed a three point grading system based on the evaluation of the number and size of blastomeres and the degree of fragmentation [3]. Racowsky et al. evaluated the system and reported a significant association with life birth [4]. However, the assessment of embryo grading has been associated with considerable intra- and inter-observer variability [5,6], due to the absence of a golden standard. Although expert opinion has been considered as the golden standard in a multicentre study [5], this choice can be scientifically challenged since the level of experience is not necessarily linked to good inter-observer agreement in the assessment of embryonic multilevel images as we reported recently [6]. Embryo morphology assessment based on multilevel images has the advantage to allow an unlimited number of observations to measure intra- and inter-observer agreement, when compared to classical observation using an inverted microscope, characterized by a limitation in evaluation time.

Besides embryo morphology, a second important subject of intra- and inter-observer quality control deals with the decision on which embryo needs to be transferred, cryopreserved or discarded. This clinical decision making has been associated with moderate (Assin et al. [7] and Castilla et al. [8]) and high (Arce et al [9]) inter-observer agreement.

We propose that multilevel images, distributed via a website, can be helpful to facilitate and improve external quality control on morphological aspects of zygote and embryo and on clinical decision making. In the present multicentre study, the aim was to determine if the intra- and inter-observer agreement in the morphological assessment of human embryos using multilevel images can be measured.

## Methods

### Assessment of intra-and inter-observer agreement

A total of five embryologists of four different IVF centers (Radboud University Nijmegen Medical Centre; the Netherlands; Centre de Procréation Médicalement Assistée de l'ULg, CHR de la Citadelle, Liège, Belgium, Service PMA, Centre Hospitalier Régional de Namur, Namur, Belgium; Leuven University Fertility Centre, UZ Gasthuisberg, Leuven, Belgium) participated in this study. On a website, multilevel images (= 26 sequential images of the same oocyte or embryo by automatically focusing through the complete embryo at 5 μm intervals) of 90 embryos were presented twice to evaluate the intra- and inter-observer agreement. A total of six evaluation sessions were performed (each session containing 30 unique embryos). Each set of 30 embryos had to be completed within 2 days and the time interval of evaluation between two sets of embryos was at least 2 weeks (and at most 4 weeks) according to a given time schedule. Each embryologist was blinded with respect to the assessment of the embryo quality in his/her first evaluations and to the results of the assessments by the other embryologists.

### Website design

**Figure 1:**
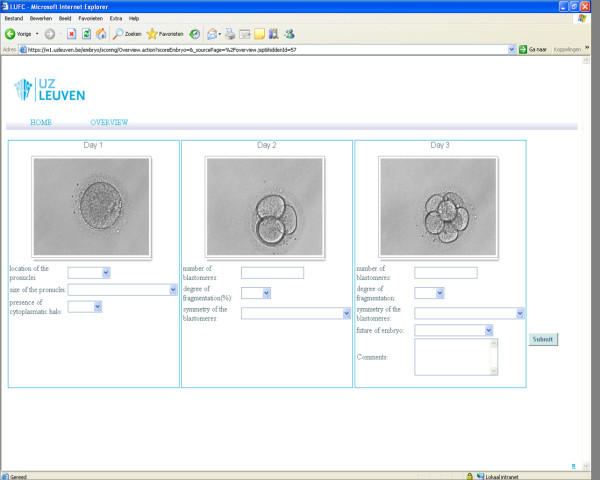
**Detailed overview of the embryos (multilevel image) on day 1, day 2 and day 3**.

Using the MoSCoW (Must have-Should have-Could have-Won't have) method a template was presented on a wiki page to discuss which information was needed on the website. A proof of concept of the website was made. The site consisted of a part for the embryologists who had to evaluate the embryos and a part for the administrator who managed the images. Each embryologist had an overview of the assigned embryos and a detailed image (multilevel) of each individual embryo on day 1, day 2 and day 3 (Figure 1). The embryologist had to enter his/her embryo score using predefined values of each of the characteristics. The administrator used a section where images of embryos were entered, deleted or updated and evaluation data could be extracted. The website was developed using Java Server Pages (JSP) in the Stripes Framework and the scoring information was stored in a SQL2008 database.

### Embryo evaluation

All zygotes and embryos for this study were derived from routine fresh IVF/ICSI treatments in the Leuven University Fertility Centre using previously described ovarian stimulation protocols [10]. Embryos were cultured in a sequential culture medium (Sydney IVF medium, COOK, Brisbane, Australia) under 5% CO2 and 20% O2 at 37°C. Multilevel images were obtained on day 1, day 2 and day 3.

In total, six sets of 30 embryos were evaluated on day 1, day 2 and day 3 of their development based on the following criteria agreed among the different centers. Day 1 embryos were evaluated based on the position and equality of the pronuclei and on the presence of a cytoplasmic halo. Day 2 and day 3 embryos were evaluated based on the number and size of their blastomeres and the degree of fragmentation (0: 0% fragmentation; 1: < 10%; 2: 10-25%; 3: 26-50%; 4: > 50% fragmentation). In addition, the embryologists had to decide if the embryo would be transferred, cryopreserved or discarded (clinical decision) on day 3. The result of embryo evaluation on day 1, day 2 and day 3 was annotated online under each multilevel image and had to be finished before a next embryo could be evaluated.

### Statistics

The Cohen's kappa coefficient was calculated to measure intra-observer (comparison of embryo scoring given at two different time points by the same embryologist) and inter-observer (comparison of embryo scoring by different embryologists) agreement. The kappa value standardized to lie on a -1 to 1 scale where 1 is perfect agreement and 0 represents what would be expected by chance. Negative values indicate agreement less than chance with a potential systematic disagreement between the observers [11]. This kappa coefficient was interpreted as an indicator of either excellent (≥0.80), good (0.60-0.79), moderate (0.40-0.59), poor (0.20-0.39) and very poor (< 0.20) intra- and inter-observer agreement [12]. The number of observations necessary to do kappa statistics is calculated by the equation: 2n^2^; with n the number of categories for each characteristic. In this study, the degree of fragmentation has the highest number of categories (n = 5) indicating the need for at least 2*(5)2 = 50 embryos [13]. A total of 90 embryos were included in this study.

## Results

The results of the intra-and inter-observer agreements are shown in table 1 and table 2 respectively.

**Table 1:** Intra-observer agreement indicated by the median (range) value of the kappa coefficient

Characteristics	Median kappa coefficient (range)
**Characteristics day 1**	
Position of the pronuclei	0.74 (0.65-0.77)
Size of the pronuclei	0.44 (0.33-0.57)
Cytoplasmic halo	-0.34 (-0.62-0)
**Characteristics day 2**	
Number of blastomeres	0.82 (0.80-0.93)
Degree of fragmentation	0.58 (0.53-0.62)
Size of blastomeres	0.59 (0.48-0.62)
**Characteristics day 3**	
Number of blastomeres	0.67 (0.57-0.82)
Degree of fragmentation	0.59 (0.51-0.65)
Size of blastomeres	0.55 (0.47-0.76)
**Decision**	0.75 (0.72-0.88)

**Table 2:** Median (range) value of the kappa coefficient as measurement for the inter-observer agreement

Characteristics	Median kappa coefficient (range)
**Characteristics day 1**	
Position of the pronuclei	0.66 (0.37-0.86)
Size of the pronuclei	0.16 (0.09-0.27)
Cytoplasmic halo	-0.16 (-0.32-0.05)
**Characteristics day 2**	
Number of blastomeres	0.73 (0.71-0.83)
Degree of fragmentation	0.46 (0.20-0.61)
Size of blastomeres	0.32 (0.11-0.50)
**Characteristics day 3**	
Number of blastomeres	0.63 (0.57-0.74)
Degree of fragmentation	0.49 (0.20-0.57)
Size of blastomeres	0.39 (0.13-0.57)
**Decision**	0.71 (0.67-0.86)

The median kappa coefficients for the intra-observer agreement varied over the different characteristics between -0.34 and 0.82. Good to excellent agreement was observed for the position of the pronclei, the number of blastomeres on day 2 and day 3 and the clinical decision making. Other characteristics showed poor to moderate intra-observer agreement.

For the inter-observer agreements, the same results as in the intra-observer analysis were found, with slightly lower kappa values (-0.16-0.73).

In both cases, the clinical decision scored relatively high. The most problems were found in the characteristics of the pronuclei. The position of the pronuclei showed a good agreement, however both pronuclear size and cytoplasmic halo lead to the lowest kappa values (intra-observer agreement: 0.44 and -0.34; inter-observer agreement: 0.16 and -0.16).

## Discussion

This multicentre study used for the first time multilevel images to report the intra- and inter-observer variability in the embryo evaluation. The use of these multilevel images allows embryologists to assess the embryo quality similarly as an exploration by using an inverted microscope.

The results showed a good to excellent intra-observer agreement for the evaluation of the position of the pronuclei on day 1, the number of blastomeres on day 2 and day 3 and the clinical decision. These results confirmed the results of our monocentre study [6] and the results found by Arce et al. in a multicentre trial using 2D images [9]. In contrast to our current observations, these two studies [6,9] reported also a good to excellent agreement for other characteristics (degree of fragmentation and size of blastomeres on day 2 and/or day 3). This can be due to differences in study design (monocenter study [6] and 2D images [9]).

Good to excellent inter-observer agreement was found for the evaluation of the position of the pronuclei on day 1, the number of blastomeres on day 2 and day 3 and the decision on final destiny of each embryo. This confirms the results reported in our monocenter study [6] and those published by Arce et al. [9]. In contrast, other investigators (Bendus et al. [5]; Castilla et al. [8]) reported a moderate to excellent agreement for the embryo grading on day 3. However, only supernumerary embryos were used by Bendus et al [5]. In addition, different scoring systems were used by the centers included in these studies [5,8]. Moreover, agreement on a embryo score (optimal, moderate and poor, based on the combination of different individual characteristics) was measured whereas in our study individual embryo characteristics were evaluated. In our opinion, the use of supernumerary embryos [5] or selecting embryos for the determination of intra- and inter-observer variability based on the embryo score [8] is not fully representative for the routine embryo population. Therefore, in our study, embryos from routine practice were evaluated to have a representative dataset of the daily practice. Regarding the decision making process, a good agreement was found in our study. However, other investigators (de Assin et al. [7]; Castilla et al. [8]), reported moderate agreement in the clinical decision on final destiny of each embryo. This can be due to differences in the study design. In our study, embryologists were asked to decide for each embryo if the embryo would be transferred, cryopreserved or discarded. In the studies of de Assin et al [7] and Castilla et al. [8] two embryos, from a batch of embryos per patient, needed to be selected for transfer.

A moderate to poor inter-observer agreement was reported for the evaluation of the size of the pronuclei, the degree of fragmentation on day 2 and day 3 and for the evaluation of the symmetry of blastomeres on day 2 and day 3, which is in line with the results of our monocentre study [6] and the studies of Arce et al. and Bendus et al. [5,9].

## Conclusions

Quality control, mono- or multicentre, on embryo scoring can easily be performed through the design of a simple website. In the future the website design can be adapted to generate statistical feedback upon scoring and can even include a training module. Intra-observer and inter-observer agreement in this multicentre trial was good to excellent for the position of the pronuclei on day 1, the number of blastomeres on day 2 and day 3 and the clinical decision.

## Competing interests

The authors declare that they have no competing interests.

## Authors' contributions

GP and CS contribute to the paper by defining the design of the of the study, the analysis and the interpretation of the data. Both authors draft the paper and approved the final version. SD and TD interpreted the data and revised the paper critically for important intellectual content and approved the final version. AW, TF, AvS, DW and JD revised the paper critically and added significant information and approved the final version. All authors read and approved the final manuscripts
